# Feasibility, safety and effectiveness of prednisolone and vitamin B1, B6, and B12 in patients with post-COVID-19-syndrome (PreVitaCOV) – protocol of a randomised, double-blind, placebo-controlled multicentre trial in primary care (phase IIIb)

**DOI:** 10.1186/s12879-023-08925-2

**Published:** 2024-01-06

**Authors:** Caroline Tengelmann, Stefanie Joos, Yvonne Kaußner, Uwe Malzahn, Laura Lunden, Andreas Klug, Karl Georg Häusler, Catharina Escales, Walter Maetzler, Klemens Hügen, Oliver Zolk, Peter U. Heuschmann, Christian Förster, Hanna Kaduszkiewicz, Ildikó Gágyor

**Affiliations:** 1 Department of General Practice, University Hospital Würzburg, Josef-Schneider-Straße 2, Building D7, 97080 Würzburg, Germany; 2 Institute of General Practice and Interprofessional Care, University Hospital Tübingen, Osianderstrasse 5, 72076 Tübingen, Germany; 3 Clinical Trial Center, University Hospital Würzburg, Josef-Schneider-Straße 2, Building D7, 97080 Würzburg, Germany; 4 Institute of General Practice, University of Kiel, Michaelisstraße 5, 24105 Kiel, Germany; 5 Department of Neurology, University Hospital Würzburg, Josef-Schneider-Straße 2, Building B1, 97080 Würzburg, Germany; 6 Department of Neurology, University Hospital Schleswig-Holstein, Campus Kiel, Arnold-Heller- Str. 3, Haus D, 24105 Kiel, Germany; 7 Institute of Clinical Pharmacology, Brandenburg Medical School, Faculty of Health Sciences Brandenburg, Immanuel Klinik Rüdersdorf, Seebad82/83, 15562 Rüdersdorf, Germany

**Keywords:** Post-COVID-19-Syndrome, Prednisolone, Vitamin B, Primary care, Randomised controlled trial

## Abstract

**Background:**

After infection with SARS-CoV-2 a relevant proportion of patients complains about persisting symptoms, a condition termed Post-COVID-19-syndrome (PC19S). So far, possible treatments are under investigation. Among others, neurotropic vitamins and anti-inflammatory substances are potential options. Thus, the PreVitaCOV trial aims to assess feasibility, safety, and effectiveness of treating patients in primary care with prednisolone and/or vitamin B1, B6 and B12.

**Methods:**

The phase IIIb, multi-centre randomised, double-blind, and placebo-controlled PreVitaCOV trial has a factorial design and is planned as a two-phase approach. The pilot phase assessed feasibility and safety and was transformed into a confirmatory phase to evaluate effectiveness since feasibility was proven. Adult patients with PC19S after a documented SARS-CoV-2 infection at least 12 weeks ago are randomly assigned to 4 parallel treatments: prednisolone 20 mg for five days followed by 5 mg for 23 days (trial drug 1), B vitamins (B1 (100 mg OD), B6 (50 mg OD), and B12 (500 µg OD)) for 28 days (trial drug 2), trial drugs 1 and 2, or placebo. The primary outcome of the pilot phase was defined as the retention rate of the first 100 patients. Values of ≥ 85% were considered as confirmation of feasibility, this criterion was even surpassed by a retention rate of 98%. After transformation, the confirmatory phase proceeds by enrolling 240 additional patients. The primary outcome for the study is the change of symptom severity from baseline to day 28 as assessed by a tailored Patient Reported Outcomes Measurement Information System (PROMIS) total score referring to five symptom domains known to be typical for PC19S (fatigue, dyspnoea, cognition, anxiety, depression). The confirmatory trial is considered positive if superiority of any treatment is demonstrated over placebo operationalised by an improvement of at least 3 points on the PROMIS total score (t-score).

**Discussion:**

The PreVitaCOV trial may contribute to the understanding of therapeutic approaches in PC19S in a primary care context.

**Trial registration:**

EudraCT: 2022-001041-20. DRKS: DRKS00029617. ClinicalTrials.gov: F001AM02222_1 (registered: 05 Dec 2022).

**Supplementary Information:**

The online version contains supplementary material available at 10.1186/s12879-023-08925-2.

## Background

Post-COVID-19 syndrome (PC19S) is a new pathologic entity affecting a considerable portion of patients after SARS-CoV-2 infection [1–5]. Published prevalence estimates show an enormous range between 2.3 and 28.5% which can be attributed to varying definitions of PC19S, investigated populations, considered viral variants, and methods of symptom assessment [6, 7]. PC19S is characterised by a broad range of persistent disabling symptoms and signs [8]. The most frequent symptoms include fatigue, post-exertional malaise, cognitive dysfunction, impaired attention, dry cough, shortness of breath, headache, muscle ache, chest tightness, and sore throat [9]. Most patients seek help in ambulatory care and 76% exclusively consult their general practitioner [10]. Evidence regarding specific medical treatment is sparse. Specifically, there is a lack of swiftly initiated trials that explicitly take place in primary care.

Various pathophysiological hypotheses, e.g. long-term tissue damage and chronic auto-inflammation are discussed for PC19S [11]. Preliminary findings suggest that the cytokine signature differs between PC19S and convalescent COVID-19 patients without PC19S, as found in an Italian observational study with 551 patients [12]. Furthermore, an increased demand for methyl groups, a cytokine storm and oxidative stress are discussed as potential mechanisms of PC19S [13]. Accordingly, patients with PC19S may benefit from methyl-groups by supplementation of vitamin B12. Others suggest the supplementation of vitamin B1, B6, and B12 due to their neurotropic effects considering that most PC19S patients suffer from fatigue or other neurologic symptoms [14].

Drugs with anti-inflammatory properties such as corticosteroids, among others, were also suggested to alleviate symptoms [11]. This approach is based on the consideration of chronic inflammation, as has already been postulated for various autoimmune diseases [15] and PC19S [16]. An observational study with 30 PC19S patients showed that treatment with corticosteroids at high initial dose (26.6 mg prednisolone on the average with a maximum initial dose of 0.5 mg/kg) and a rapid wean over a few weeks was well tolerated and associated with improvement of pulmonary symptoms [17]. In another observational study, 24 patients diagnosed with Long-COVID were treated with corticosteroids in tapering doses for 8 to 10 weeks. The study reports improvement in fatigue and breathlessness, but did not include a control group [18]. A Spanish study treated 8 patients with 30 mg prednisolone for a total of 4 days. While the focus was on normalisation of immunologic laboratory findings, clinical symptoms were reported to have improved after four days [19].

Therefore, we have chosen vitamin B1, B6, and B12 and corticosteroids as therapeutic approach in PC19S patients. These drugs have been authorised for years, are successfully used for many clinical conditions with symptoms similar to PC19S, and can easily be handled in primary care including dealing with possible side effects.

Following an adaptive design, the PreVitaCOV trial comprises the pilot phase which aimed to demonstrate feasibility and safety of this pragmatic randomised controlled trial (RCT) in primary care and the confirmatory phase which shall prove the efficacy of the treatment.

By involving general practitioners (GPs) and health care professionals in university hospitals, the trial team will establish proceedings for future RCTs and provide a use case for existing research infrastructures such as practice-based research networks.

## Methods

### Trial design

The “PreVitaCOV” trial is a multicentre, double-blind, randomised and placebo-controlled phase-IIIb-trial with a factorial design (four parallel groups with a 1:1:1:1 ratio) to test superiority of prednisolone and/or the vitamin B compound over placebo. For the present trial protocol, the SPIRIT reporting guidelines were applied (supplement) [20]. It refers to the amended version 2.0 of the extended protocol approved by the ethics committees of Würzburg, Kiel and Tübingen as well as the competent authority (05 June 2023). Trial protocol and information sheets in their original and amended versions were made available to personnel involved in the PreVitaCOV trial after approval.

### Trial objectives

#### Primary outcomes

A separate primary outcome is defined for each phase of the trial:

1) For the pilot phase: feasibility of screening and recruitment in primary care as defined by the retention rate of the first 100 patients until the end of treatment on day 28. A retention rate of at least 85% was considered as confirmation of feasibility justifying transformation into the confirmatory phase. This criterion was surpassed by an overall retention rate of 98% during the pilot phase.

2) For the confirmatory phase: change of symptom severity as assessed by the Patient Reported Outcomes Measurement Information System (PROMIS) total score from baseline to day 28 [21]. The confirmatory trial will be considered positive if superiority of at least one treatment is demonstrated over placebo.

#### Secondary outcomes

If the pilot phase had not allowed a confirmatory trial, the above mentioned PROMIS total score would have been a key secondary outcome of the pilot phase. For both, the pilot and the confirmatory phase the following secondary outcomes with reference to effectiveness will be assessed: change of severity and quality of PC19 symptoms as measured by the following instruments: (1) the PROMIS subscores in the domains included in the total score [21], (2) Measure Yourself Medical Outcome Profile (MYMOP®) [22]; (3) PC19S functional status (PCFS - Scale) [23], (4) PC19S symptom list (5) health related quality of life (European Quality of Life 5 Dimensions 5 Level Version (EQ-5D-5 L) and visual analogue scale for quality of life) [24], (6) The eight-item Patient Health Questionnaire depression scale (PHQ8-depression scale) [25], (7) Chalder Scale for fatigue [26], (8) numeric rating scale for pain. In addition, the change of cognitive functions is objectively assessed by performing a battery of tests to assess alertness, divided attention, distractibility, visual scanning and flexibility as subtypes of attention according to Zimmermann & Fimm [27]. Change of physical fitness is assessed by the 1-minute Sit-to-Stand-Test [28]. Also, data on the use of on-demand medication are collected. With reference to feasibility and acceptance, a survey at the end of data collection after six months is performed. Additionally, qualitative interviews on views and perspectives regarding care are carried out with a sub-group sample of five to seven patients per trial site.

With regard to safety, various outcomes are considered: e.g., change in the results of the physical examination, number of patients with worsening symptoms, number of adverse events (AEs) and severe adverse events (SAEs) by system organ class and proportion of patients with at least one AE within two months after enrolment.

#### Sample size

Sample size calculation for the pilot phase was driven by the aim to ensure the estimation of the retention rate with sufficient precision (width of maximal 0.15, i.e.15%, of the corresponding 95% confidence interval). Based on the exact (Clopper-Paerson) method with the assumption of an observed sample proportion of 0.85 (85%) for the target retention rate, enrolment of 100 patients was necessary. According to the trial design, four equally sized treatment groups (4 * 25 = 100 patients) were planned.

The aim of the sample size calculation for the confirmatory phase is the detection of an underlying main effect that corresponds to at least 3-points (T-score) on the PROMIS outcome scale, assuming a difference in T-score of at least 3 points as a reasonable minimally important difference (Kroenke [21] et al.). The primary outcome scale is a tailored weighted mean T-score from the five PROMIS subscales, ranging from 16.7 to 57.7 [21]. Within the primary analysis, the four treatment groups will be compared: patients with intake either of placebo, vitamin B compound only, prednisolone only or both vitamin B compound and prednisolone combined.

We will estimate the main and interaction effects and test the three null hypotheses of no treatment effect of the main factor vitamin B compound, no treatment effect of the main factor prednisolone, and the null hypothesis of no interaction between the factors vitamin B compound and prednisolone. The T-score difference of 3 points corresponds to a standard deviation of means (SM) of 1.5. The estimated sample size of 4*72 = 288, based on a two-factor analysis of variance (ANOVA), ensures that any main treatment effect corresponding to a SM of at least 1.5 (vitamins B1, B6 and B12 and prednisolone) can be detected with sufficient power (80%) at a significance level of α = 0.05. Assuming a lost to follow up rate of 15%, 4*85 = 340 patients are to be included in the trial.

### Trial population

#### Setting and recruitment

The multicentre trial is conducted with a sample of patients recruited via primary care just as on the patient’s own initiative.

Patients who consult their GP with symptoms of PC19S are pre-screened by GPs in their practice. In addition, GPs may identify suitable patients based on their electronic health records. GPs are asked to refer eligible patients to either of the three trial sites located at the Departments of General Practice at the medical faculties Würzburg, Tübingen, and Kiel. Further recruitment strategies include social media and press releases.

#### Prescreening and screening

Patient pre-screening follows a pragmatic approach using the GPs’ experience in patient assessment in terms of eligibility for our trial mimicking a real-life situation. Patients who contact one of the trial sites directly must present to his/her GP in advance, so that the GP can refer important medical data (such as medical history, concomitant medication) and confirm the diagnosis of PC19S.

#### Inclusion and exclusion criteria

Potentially eligible patients are carefully re-assessed by clinicians at the trial site at day 0 (baseline) before the patient’s enrolment according to the following in- and exclusion criteria:

##### Inclusion criteria

(1) adult patient, (2) history of documented SARS-CoV-2 infection at least 12 weeks ago (with reference to the infection causing the PC19S), (3) symptoms concerning at least one of the following domains: fatigue, dyspnoea, cognition, anxiety, depression, and (4) symptoms that developed during or after the SARS-CoV-2 infection, persist until trial inclusion and are associated with COVID-19 as assessed by the patient’s GP or local investigator.

##### Exclusion criteria

(1) acute COVID-19 infection at baseline visit (assessed by SARS-CoV-2 antigen test), (2) patients who were treated in the intensive care unit because of COVID-19, (3) pregnancy/breastfeeding (4) diabetes mellitus, (5) hypertension, (6) possible explanation of PC19S symptoms by alternative diagnoses (e.g., chronic fatigue syndrome, depression, active or preceding cancer therapy, severe anaemia, sleep apnoea syndrome) as assessed by the patients’ GP or the investigator, (7) history of severe medical conditions (such as concomitant acute infectious disease, gastrointestinal ulcer, liver disease/liver cirrhosis, malabsorption or condition after bariatric surgery, chronic airway disease [e.g., asthma, COPD], chronic heart failure [NYHA III or IV], neurologic disorders [e.g., multiple sclerosis, motoneuron disease], untreated hypothyroidism, significantly impaired glucuronidation [e.g., Gilbert-Meulengracht, ROTOR, or Crigler-Najjar syndrome], immunodeficiency or a chronically weakened immune system [e.g., HIV, AIDS, lymphoma, chemo-radio-therapy, immunosuppressive pathology], mental disorders [e.g., depression, psychosis, dementia], active cancer, or any other severe medical conditions that preclude participation as determined by the responsible physician), (8) current use of immunosuppressive drugs, non-steroidal anti-inflammatory drugs [NSAIDs], fluoroquinolones, anticoagulation (phenprocoumon or other cumarin derivates, direct oral anticoagulants), acetylsalicylic acid (ASA), or any other drug with a possible and clinically relevant interaction with the trial medication, (9) current or previous treatment with any of the trial drugs for at least seven days since COVID-19 or any parenteral application (includes vitamin supplements containing vitamin B1, B6, or B12), (10) known allergy and contraindications to the intervention drugs, (11) need of care and/or peer dependency, (12) nursing home residents, (13) inability to understand the scope of the trial, to follow trial procedures and to give informed consent or to attend the trial sites, (14) participation in another interventional trial at the same time or within the past three months before enrolment, or (15) female patients considering to get pregnant during the trial and within one week after the last dose of the trial drug(s).

During pre-screening for the first trial subjects, it became apparent that three criteria hindered the inclusion of potential subjects. Therefore, we adjusted them in an amendment:

Inclusion criterion 2: Many of the eligible subjects were no longer able to provide a PCR test as a proof for a passed SARS-CoV-2 infection, due to the change in regulatory requirements and the more pragmatic approach to this condition. After the amendment, inclusion is now possible based on submission of a valid PCR or antigen test or antibody test in combination with a matching clinical history as confirmed by the patient’s GP.

Exclusion criterion 5: In a considerable number of potentially eligible patients, hypertension is present. The exclusion of these patients would have meant, that the overall population was not represented in this trial. After weighting the low risk of hypertensive derailment due to trial medication, we decided to include these patients under close-meshed self-monitoring of blood pressure within the first week of study participation.

Exclusion criterion 9: Many of the principally suitable patients have already undertaken therapy attempts with B vitamins independently from or according to the recommendation of their treating physician. The intake of any dose had been an exclusion criterion in the first version of the protocol. We adapted this in the amendment to any parenteral administration or intake of vitamins B1, B6, B12 in a dose equivalent to the trial medication for at least seven days. In case of ingestion of lower doses, the intake should be ceased at least four weeks prior to trial inclusion.

#### Trial drugs

Prednisolone is applied as *Trial drug 1* for five days with a daily oral dosage of 20 mg and subsequently for 23 days with a dosage of 5 mg (both one capsule/day). At first it was distributed by the Hospital Pharmacy of the Charité-Universitätsmedizin Berlin. For organizational reasons, responsibility for the production of trial drug 1 and its placebo was transferred from the Hospital Pharmacy of the Charité – Universitätsmedizin Berlin to the pharmacy of the University Hospital Hamburg-Eppendorf as from march 2023.

Prednisolon STADA® 10 mg and Predni H Tablinen® Zentiva 5 mg tablets are encapsulated and packed into a box containing 5 capsules of prednisolone 20 mg and a second box containing 28 capsules of prednisolone 5 mg.

The Vitamin B compound as *Trial drug 2* contains vitamins B1 (100 mg), B6 (50 mg), and B12 (500 µg) and is provided by Hevert-Arzneimittel GmbH in bulk blisters à 20 film coated tablets. Two blisters were packaged in folding boxes by the Hospital Pharmacy of the Charité – Universitätsmedizin Berlin, Germany.

Trial drugs 1 and 2 are combined either with a placebo for the vitamin B compound or a placebo for prednisolone (one capsule/day), which are provided by Hevert-Arzneimittel GmbH and the Hospital Pharmacy of the Charité respectively and packed in the same manner as the trial drugs themselves.

Trial drugs 1 and 2 are combined as *Treatment Arm 3*. Placebo as *Treatment Arm 4* is used as a comparator not only to overcome context effects of the trial but also to balance for placebo effects. Yet, the use of placebo as a comparator is resulting in some patients not receiving the active treatment. However, as long as effective evidence-based treatment options are lacking, patients randomised to placebo are not deprived of the standard care.

All blisters and boxes are labelled in a blinded manner. Table 1 provides an overview of the trial drugs and the duration and dosage of intake.

After written informed consent and random assignment to either of the treatment groups, patients receive the trial drugs as well as instructions on handling and storage. Drug accountability is performed according to GCP-Guidelines.

**Table 1:** Treatment arms, duration and dosage of intake

	Day 0	Day 1–5	Day 6–28
**Treatment Arm 1** (prednisolone and placebo)	enrolment	20 mg prednisolone 1 × 1 and placebo 1 × 1	5 mg prednisolone 1 × 1 and placebo 1 × 1
**Treatment Arm 2** (placebo and vitamin B compound)	enrolment	placebo 1 × 1 and Vitamin B compound (100 mg B1, 50 mg B6, 500 µg B12) 1 × 1	
**Treatment Arm 3** (prednisolone and vitamin B compound)	enrolment	20 mg prednisolone 1 × 1 and Vitamin B compound (100 mg B1, 50 mg B6, 500 µg B12) 1 × 1	5 mg prednisolone 1 × 1 and Vitamin B compound (100 mg B1, 50 mg B6, 500 µg B12) 1 × 1
**Treatment Arm 4** (placebo and placebo)	enrolment	placebo 1 × 1 and placebo 1 × 1	

Patients are advised to avoid the intake of drugs that may interfere with the trial medication:

(1) nonsteroidal anti-inflammatory drugs (NSAIDs), i.e., painkillers like ibuprofen or diclofenac; acetylsalicylic acid (ASA) even in prophylactic anticoagulatory dosage. This information is also provided to the study participants in form of a leaflet. If the use of NSAIDs or ASA becomes necessary, participants are advised to consult their GP.

(2) fluoroquinolones.

(3) antacids (magnesium-/aluminiumhydroxid) for at least two hours after taking the trial medication.

(4) any other drug with a possible and clinically relevant interaction with the trial medication (as described in the corresponding summaries of product characteristics (“Fachinformationen” [29–31])).

(5) the trial medication available as over the counter drugs.

#### Clinical trial procedures

At the *baseline visit (day 0)*, after informed consent, patients are randomly assigned to one of the four parallel arms via the eCRF. In women of childbearing potential, a urine pregnancy test is performed. Also, relevant data on demography and the preceding SARS-COV-2-infection, medical history, concomitant medication, mental fitness (Montreal Cognitive Assessment (MoCA [32]) and physical activity as well as baseline biosamples (Table 2) and vital signs (e.g. blood tests, blood glucose, antibody test for SARS-CoV-2, height, weight, O_2_ saturation, heart rate, blood pressure) are collected. A physical examination (e.g., auscultation of chest and heart, assessments of neurological status, oedema and lymph node status) is performed. Furthermore, baseline values of the outcomes are assessed via the above-mentioned questionnaires and tests and the blinded trial medication with instructions on intake and a patient diary are dispensed together with an emergency card.

By means of a standardised telephone interview carried out by trained staff (*phone call 1, day 5 (+ 3)*) the patients are questioned on (selected) endpoints (Table 3) to reassess symptom severity (PROMIS, MYMOP, EQ-5D-5 L, PCFS, Chalder Fatigue Scale, VAS and the PHQ questionnaire), the consumption of concomitant medication or the occurrence of (S)AEs.

The patients are asked to complete a patient diary from day 1 to day 28 noting the intake of the trial drugs and any additional medication. Furthermore, all patients are asked to monitor their blood pressure values during the first seven days of the intake of the trial medication. Moreover, the PROMIS short forms are to be filled out on day 14 and 21 by the patients themselves.

On day 28, patients present at the trial site for *visit 2* to complete the above-mentioned questionnaires and tests for the primary and secondary endpoints again. Vital signs are taken, a physical examination is carried out and selected laboratory parameters are re-assessed (Table 2). Additionally, the patient is asked about the intake of concomitant medication, the usage of additional therapies, the occurrence of (S)AEs and the remaining drugs which are documented. Women of childbearing potential receive a pregnancy test to perform by themselves at the earliest on the 4th day after the end of medication intake (day 32). Its usage is explained.

#### Follow-up phone calls

The first follow-up phone call is performed two months after enrolment (*phone call 2, day 60*), the final follow-up and close-out phone call is scheduled six months after enrolment (*phone call 3, day 180*). As during phone call 1, patients are interviewed on selected endpoints as listed in Table 3 to reassess any change of symptom severity again (as on day 5). Furthermore, they are asked about their usage of additional therapies, new medical treatment and changes of their long-term or concomitant medication and the occurrence of (S)AEs. After the close-out phone call, patients are asked to fill out a self-developed questionnaire on perception and acceptance of the trial. To avoid distortion because of social desirability, they are asked to return it by mail.

Additionally, a sub-sample of patients is interviewed before enrolment in the trial based on a semi-structured interview-guide. Questions focus on the patients’ perception of health care and their needs regarding health care in the context of PC19S. Main objectives of this qualitative sub-study are a better understanding of current post-COVID care as perceived by patients, and an identification of approaches to improve care of these patients.

#### Discontinuation of the trial

Discontinuation of the trail for (single) patients can be necessary in case of: patient’s request, withdrawal of consent, loss to follow-up, loss of contact, relocation of the patient, trial termination, significant non-compliance, retrospectively discovered failure to fulfil inclusion/exclusion criteria if individual benefit/risk assessment does not overrule, new medical conditions not allowing continuation of the medication according to the protocol, occurrence of adverse events (e.g., allergic reaction to trial medication, severe laboratory abnormalities) leading to substantial changes in the individual risk-benefit considerations that suggest a discontinuation of the trial drug, pregnancy, enrolment in any other clinical trial involving an investigational product or enrolment in any other type of medical research judged not to be scientifically or medically compatible with this trial, treatment with another therapeutic agent that has been demonstrated to be effective for treatment of the trial indication, treatment with a concomitant medication that during this trial should be avoided for reasons of safety and/or efficacy. In those cases, data collected up to this point will be used, all remaining follow-up visits and phone calls will be offered, and analysis of data will be performed as intention-to-treat analysis.

For any harm caused by participation in this trial there is a patient insurance providing insurance for trial-related injuries to health, covering any injuries, which the patient suffers directly or indirectly of as a result of the trial product or interventions connected with the clinical trial as well as a securing insurance for all patients travelling to and from the trial site.

**Table 2:** Blood and urine tests to be performed at baseline (day 0) and at visit 2 (day 28)

**After enrolment in the trial at baseline (Visit 1)** - conventional urine pregnancy test (sensitivity down to at least 25mlU/ml for human chorionic gonadotrophin (hCG) for women of childbearing potential - antigen test for SARS-CoV-2 - antibody test for SARS-CoV-2 (anti-nucleocapsid) - glutamate-oxalacetate-transaminase (GOT/ASAT) - glutamate-pyruvate-transaminase (GPT/ALAT) - gamma-glutamyltransferase (GGT) - creatinine and glomerular fitration rate (GFR) - thyroid-stimulating-hormone (TSH) - potassium, sodium, calcium - non-fasting glucose - regular and differential blood count - bilirubin - cortisol-level - vitamin B12 status (homocysteine, methylmalonic acid (MMA), holotranscobalamine) - cytokines
**At visit 2 (day 28 + 3)** - non-fasting glucose - vitamin B12 status (homocysteine, methylmalonic acid (MMA), holotranscobalamine) - cytokines
**Day 32 + 2** - conventional urine pregnancy test (sensitivity down to at least 25mlU/ml for human chorionic gonadotrophin (hCG) for women of childbearing potential

**Fig. 1:**
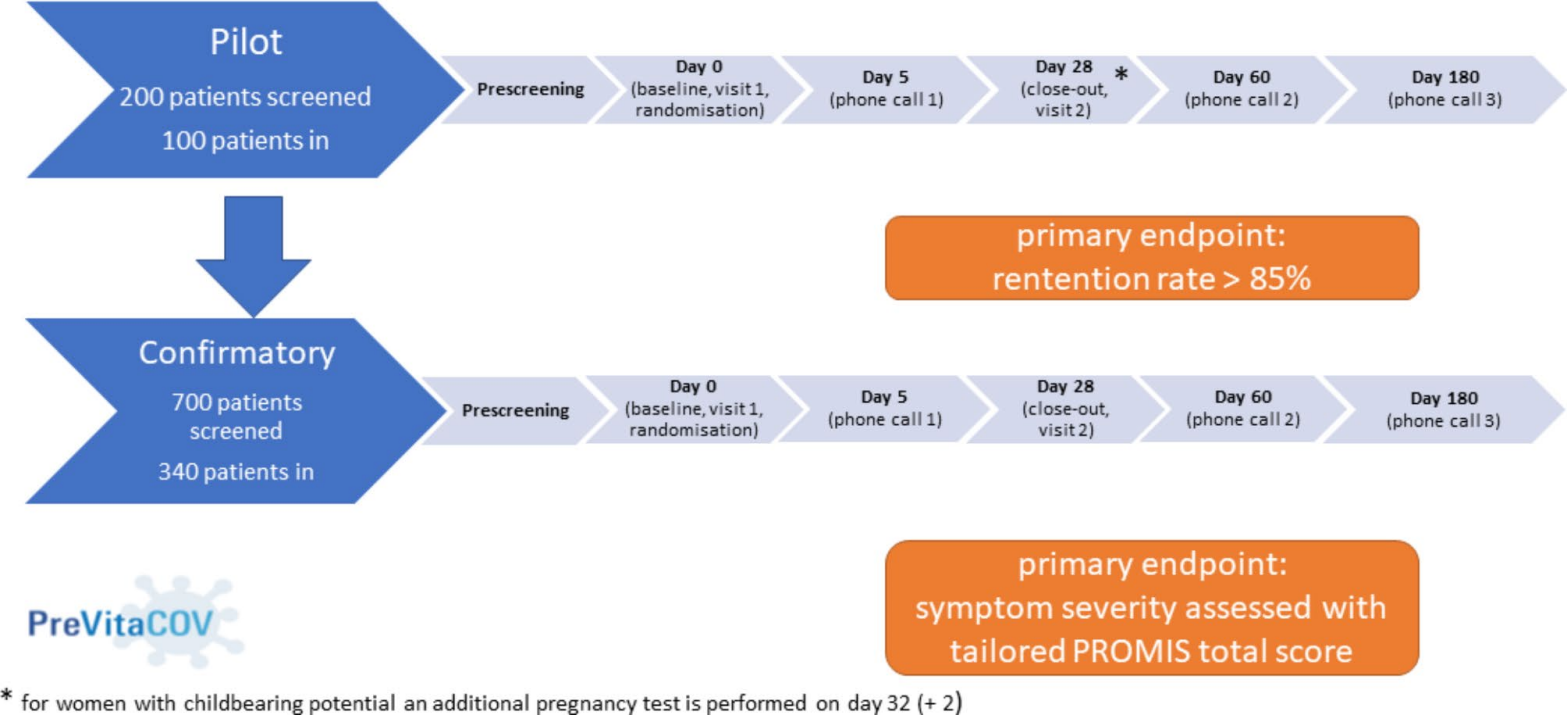
Flowchart on trial timelines

Figure 1 illustrates the design of the trial with two phases and summarises timelines per patient. Table 3 summarises visits, assessments as well as data and outcomes to be collected in a chronological order.

**Table 3:** Visit schedule, assessments as well as data and outcomes to be collected

Patient contact	Baseline	Intervention								Follow-up		
Day	0	1–4	5*	6–13	14	15–20	21	22–27	28*	32–35	60**	180***
**Visit number**	1								2			
**Phone call number**			1								2	3
**Trial drug intake**		X	X	X	X	X	X	X	X			
information, verification of in-/exclusion criteria, informed consent	X											
demographic and baseline data, medical history, MoCA	X											
biosamples, vital signs	X								X			
SARS-CoV-2 antigen test	X											
pregnancy test	X									X		
**Outcomes**												
- tailored PROMIS total score and subscores	X		X		X		X		X		X	X
- MYMOP	X		X						X		X	X
- PC19 symptom list	X		X						X		X	X
PCFS scale	X		X						X		X	X
- EQ-5D-5L and visual analogue scale	X		X						X		X	X
- PHQ 8	X		X						X		X	X
- Chalder Fatigue Scale	X		X						X		X	X
- Visual analogue scale for pain	X		X						X		X	X
- Tests Alertness, divided attention, distractibility, flexibility and visual scanning (TAP)	X								X			
- 1 min-Sit-to-Stand-Test, physical examination	X								X			
- Patient diary (on-demand medication)		X	X	X	X	X	X	X	X			
- Feasibility & acceptance questionnaire												X
- Feasibility & Acceptance Interviews (subgroup of patients)	X											
**Safety outcomes**												
- adverse events			X						X		X	X
- serious adverse events	as necessary											
- number of patients with worsening symptoms	as necessary											
Drug accountability			X						X			

* + 3 days; ** +/- 5 days; *** +/- 7 days; MoCA - Montreal Cognitive Assessment; PROMIS - Patient Reported Outcome Measurement Information System; MYMOP - Measure Yourself Medical Outcome Profile; PC19 - Post-COVID-19; PCFS - Post COVID Functional Scale; EQ-5D-5L - EuroQol-5Dimensions- 5Levels; PHQ8 - Patient Health Questionnaire 8; TAP - Test of Attentional Performance

#### Randomisation and blinding

Patients are allocated to one of the four treatment groups via stratified block randomisation with varying block size. The single stratification factor is the trial centre. Based on the randomisation list, the trial medication is labelled with code numbers.

Computerised central randomisation and generation of the randomisation list, recording code numbers and assignment to the treatments, took place at the Centre for Clinical Trials of the University Hospital Würzburg (CTCW), supervised by the trial statistician. Based on the randomisation list, the trial medication was labelled with code numbers by the Trial Pharmacy. By randomisation using the eCRF a code number is allocated to the patient, assigning him/her a certain trial medication.

The double-blind design of the trial is achieved by a double dummy design: identical appearance of the tablets containing vitamin B or placebo and identically encapsulated tablets for prednisolone or placebo.

Early unblinding is performed only in case of medical emergency when it is essential to know the trial medication, i.e., when clinical management depends importantly upon knowledge of whether the patient received prednisolone and/or vitamin B compound. This applies for pregnancies, too. Therefore, contact information is provided on the patient emergency card for each trial centre and consistent reachability is assured at any time. Unblinding is performed by using the unblinding tool integrated in the eCRF. If the eCRF is not available, unblinding must be performed manually using emergency envelopes.

In case of unblinding the trial medication will be stopped, but the patient will be monitored at least until the end of the follow-up.

#### Data management, statistical analysis and reporting

Data management, -processing as well as implementation and programming of the trial database is performed by data management of the CTCW. They developed a computerised data entry and management system (eCRF) where all trial data relating to the trial can be entered, providing the capability to perform major data management activities within a consistent, auditable and integrated electronic environment. Range, validity, and consistency checks are implemented in the system for application during data entry. In case of the necessity of corrections or data inconsistencies data queries are generated by the data manager. All corrections are traceable at any time.

After the database is locked, data will be imported into standard statistical software systems and analysed by biostatisticians at the CTCW according to a detailed statistical analysis plan (SAP). Planned statistical analyses will be done using the current versions of SAS (SAS Institute Inc., Cary, NC, USA) as well as IBM SPSS. In case of not answered items for the 5 PROMIS Short Forms – either marked by the word “SKIP” or a blank space – the free Health Measuring Scoring Service (HMSS) algorithm will be used for dealing with missing data at the calculation of T-scores. Generally, multiple imputation at the score level will be used for the patient reported outcome measures.

The *primary* *outcome* *for the pilot phase* was the retention rate at day 28 of the trial. The *primary* *outcome* *of the confirmatory phase* is a tailored, patient reported outcome for the disease burden of patients with PC19S operationalised as a weighted mean of the T-scores from five PROMIS® Short Form instruments (PROMIS Short Forms v1.0 for Fatigue (4a), Dyspnoea Severity (10a), Emotional Distress-Anxiety (4a) and Emotional Distress-Depression (4a), and PROMIS Short Form v2.0 – Cognitive Function (4a)). It is a linear combination adding up the T-scores for the four negatively worded instruments (Fatigue, Dyspnoea Severity, Anxiety, and Depression) and subtracting the T-score for the positively worded instrument (Cognitive Function). The primary outcome measure is defined as the difference in the total score from baseline to visit 2 (day 28 + 3). Assuming a medium strength of correlation between measurements at baseline and visit 2, a SD between 7 and 8 points can be assumed for this difference.

The PROMIS total score will be analysed by (1) summarising descriptive statistics at baseline and day 28 (visit 2) within the four treatment groups, (2) mean change from baseline to day 28 with 95% CI within the four treatment groups, (3) factorial analysis of variance (ANOVA) with the dependent variable change from baseline to day 28 and two independent variables (treatment with prednisolone yes vs. no, treatment with vitamin B compound yes vs. no) regarding both their main effects and their interaction. In exploratory analyses, factors potentially influencing the primary outcome, such as trial centre, age, symptom duration or laboratory parameters, will be investigated using appropriate multivariable modelling. Also, the numbers of patients with worsening symptoms will be calculated.

For all *secondary outcomes* the corresponding total sum scores and if applicable subscores will be calculated for the different visits together with their change from baseline to day 28. For selected secondary outcomes analyses will be carried out for the corresponding change as for the primary outcome by a 2 × 2 factorial ANOVA. Where appropriate, convenient transformations will be made. Furthermore, all secondary outcomes will be analysed presenting the appropriate summarizing descriptive statistics by study groups.

*Safety outcomes* will be analysed by reporting frequencies (percentages) of AEs and SAEs as well as the number of patients with worsening symptoms for each group.

An *interim analysis regarding the retention rate* was performed to decide about the transformation of the pilot phase into a confirmatory phase. The retention rate of 0.98 surpassed the predetermined benchmark of 0.85 (85% of 100 enrolled patients) for being considered sufficient. Secondary outcomes were not analysed at this time and patients enrolled in the pilot phase will be included in the final analyses of the confirmatory study.

#### Patient safety

At inclusion, patients are advised to consult their GP in case of ongoing or worsening symptoms. Patients are asked about the occurrence of (S)AEs during visits to the trial centre as well as during telephone visits from day 0 till day 180 (patient close-out). Every (S)AE is documented in the eCRF. The investigator must inform the sponsor without delay, but not later than 24 h after becoming aware of the occurrence of any SAE. Additionally, the DSMB will regularly assess safety risks based on the safety related data. In case of a cumulative occurrence of SAEs, the DSMB will evaluate the situation and recommend whether to continue or terminate the trial.

#### Ethical considerations

Ethical approval has been obtained by the Independent Ethics Committees of the University Hospital Würzburg (No. 110/22_ff), the University Hospital Tübingen (No 450/2022AMG2), and the University of Kiel (No. B 265/22/RE). Informed written consent is obtained from all patients prior to any trial-related procedure. The trial is conducted according to the principles of Good Clinical Practice (GCP) and the principles of the Declaration of Helsinki.

This study is ethical, because there are legitimate chances of benefit from the study treatment, which are offset by only limited risks of harm known from long-term experience with the study drugs. A comparison with placebo is necessary for compelling and scientifically sound methodological reasons and ethically justified because there is no effective evidence-based option for the treatment of PC19S to date. Therefore, patients randomly assigned to the placebo group will not be deprived of standard care. Careful selection of the inclusion and exclusion criteria, as well as precise examination before inclusion of the patient, ensure the lowest possible risk for the individual patient.

Close contact during the period of the intake of the trial medication makes it possible to detect a significant worsening of the symptoms at an early stage. The patient has the possibility to terminate the participation in the trial at any time.

The individual study participant cannot expect any personal health benefit, only the prospect of benefit to the population of PC19S patients, as neither efficacy nor allocation to the verum group can be guaranteed.

#### Patient involvement

The citizens’ forum, an open format at the Department of General Practice at the University of Würzburg to facilitate engagement of the public in primary care research (www.allgemeinmedizin.uni-wuerzburg.de/forschung/buergerforum/), is being actively involved in the project. In a first meeting (April 2022) we introduced the trial, asked to review recruitment material, and discussed the selection of in- and exclusion criteria with 5 patients. In a second meeting (July 2022) 10 patients were asked to review the patient diary, questionnaires, and the patient information. We have carefully considered their comments on comprehensiveness, e.g., regarding the wording of the information letters, and revised the material accordingly. Another presentation on April 2023 was used to discuss new possible recruitment strategies. In future meetings we will share experiences in recruiting patients and present our results.

#### Safety board/steering committee

An independent Data and Safety Monitoring Board (DSMB) is established to ensure safety and data quality by considering adherence to protocol and monitoring reports including amount and seriousness of findings. It will advise whether to continue, modify, or stop the trial, review AEs, SAEs and Suspected Unexpected Serious Adverse Reactions (SUSARs). The committee comprises independent board members including a statistician, a pharmacist and epidemiologist, a GP and a specialist on clinical infectiology. The board is planned to meet on a regular basis at least once a year.

#### Registration

The trial is registered at EudraCT (2022-001041-20), the German clinical trials register DRKS (DRKS00029617) and ClinicalTrials.gov (F001AM02222_1 registered: 05 Dec 2022).

#### Auditing

Clinical monitoring, audits, and inspections, if requested by authorities, are being conducted during the clinical trial for quality assurance purposes. Monitoring is carried out by a monitor from the CTCW. An adaptive monitoring plan will be implemented following any specific risk assessment. The scope and contents are laid down in a trial-specific work instruction. On-site monitoring visits were performed at the beginning of the trial and planned in the further course for source data verification and to ensure correct procedures and documentation.

In case of a trial sites audit, initiated by the sponsor, the supervisory authorities, the registration authorities, or a member of the responsible ethics committee, the clinical investigator will allow the auditor as well as the monitor access to all premises used for trial execution and documentation of the clinical trial.

## Discussion

This multicentre, placebo and randomised controlled trial with a factorial design investigates the treatment of patients with PC19S with prednisolone and/or a vitamin B compound for 28 days in primary care.

### Trial objectives

The trial follows an adaptive design involving two phases and pursues two major objectives, accordingly: First, feasibility of a RCT in primary care, demonstrated by a retention rate of ≥ 85% in 100 enrolled patients until day 28. Since feasibility was given, the trial was transformed into a confirmatory phase to assess the efficacy of the treatment in a total sample size of 340. A range of secondary outcomes and (S)AEs and worsening symptoms are assessed to explore efficacy and safety in more detail.

### Trial drugs

Like all corticosteroids, prednisolone is a potent anti-inflammatory drug used in a variety of auto-inflammatory and rheumatoid conditions. Most effective is a pulse therapy regimen with high initial doses. To achieve a good balance between therapy efficacy and potential side effects, we decided to administer prednisolone during the first five days at a dose of 20 mg, considered as an intermediate dose [33]. Due to its rapid anti-inflammatory effect, changes in clinical manifestations can usually be observed within a few days. The regimen continues with a dose of 5 mg after day five in order to maintain the anti-inflammatory effect after the initial induction. Recently, results were published showing that patients suffering from PC19S have significantly decreased levels of serum cortisol. Therefore a further consideration regarding the administration of prednisolone is the potential effect in terms of substitution [34].

As a further treatment option, we are testing the administration of a vitamin B complex equivalent to the supplementation of respective vitamin deficiencies [31].

PC19S is a new entity and therefore only few placebo-controlled trials are available yet. Based on the current literature [37–39] and evidence from similar conditions, the placebo response rate in patients suffering from PC19S is expected to be between 20% and 40%. However, since there is no standard therapy yet, patients receiving just placebo are not deprived of it.

### Trial procedures and outcome measures

Basically, the trial is following a pragmatic approach by including patients (re-)consulting their GP after a SARS-CoV-2-infection with persisting symptoms. The inclusion and exclusion criteria were chosen as pragmatically as possible to correspond to everyday life in general practices and to ensure good external validity. It is beyond the capacity of the trial to explicitly exclude all possible differential diagnoses by means of technical procedures that are costly and elaborate; instead, reliance is placed on the judgment of the GP who knows the patient’s history and who has performed tests to rule out alternative diagnoses.

After reviewing the literature and carefully considering both pragmatic and theoretical aspects, the specifically tailored PROMIS total scores were selected to assess the severity and symptoms of PC19S. By additional use of some of the above-mentioned instruments as secondary outcomes, comparisons regarding their sensitivity with regard to PC19S symptoms and recommendations for future (therapy) trials on PC19S will be made.

Moreover, tests are included to objectify the subjective assessments of the patients’ standardised performance, i.e. the 1-minute Sit-to-Stand-Test to proof physical fitness [28] and the TAP [27] to evaluate a profile of cognitive resp. attentional functions. As the MoCA [32] test rather addresses severe cognitive impairment or dementia, a response to our treatment visible on the MoCA seems unlikely. Therefore, it is only applied at baseline for characterisation of our sample. However, it is expected that the use of the TAP will provide a differentiated profile of the various subtypes of attention problems in PC19S patients according to accepted neuropsychological theories.

### Patient safety

Over the course of four weeks, patients will receive either prednisolone and/or a vitamin B complex and/or placebo. Patients with pre-existing medical conditions or intake of medication that could potentially interfere in a clinically relevant way with the trial medication are excluded from the trial. Careful pre-screening by the patients’ GPs and revaluation by the investigators are performed to avoid incompatibilities and drug interactions.

Great attention is paid in particular to the hypertensive and hyperglycaemic potential of prednisolone. Therefore, blood pressure and blood glucose are documented at baseline. According to the amendment, inclusion of patients with pre-existing hypertension is possible. As an additional safety measure, all patients are instructed to self-monitor their blood pressure daily for the first seven days of medication intake. A CE-certified measuring device is therefore provided to the patients at their baseline visit. Patients are instructed on how to gradually take action if their blood pressure exceeds certain limits. Finally, on the day of the second visit at the trial centre blood glucose and blood pressure are conclusively assessed.

Due to possible teratogenic potential of the corticosteroid a pregnancy test is performed in all women of childbearing potential at baseline and also four days after cessation of trial medication. In case of a pregnancy the usage of the trial drug must be discontinued immediately. If an infection with SARS-CoV-2 occurs during day 0 to 5 of the study, patients will be advised to stop the intake of the study medication. If any other study-specific events occur, in addition to being documented as an AE/SAE as usual, they will be reported promptly to the PI who will decide on clinical management. Also, an emergency contact is available 24/7 at all trial sites in the event of an emergency or the need to unblind the treatment group assignment. If SAEs or SUARs occur, the sponsor must be notified without delay, at the latest 24 h after becoming aware of it. Any SUSAR must be reported to the BfArM.

The selected dosage of the trial medication is well tested for various rheumatic and (auto)inflammatory diseases and is usually well tolerated. Side effects are usually temporary and no negative long-term effects are expected. We estimate that adverse drug events will occur less frequently with placebo or vitamin B complex than with prednisolone.

## Conclusions

The trial confirms that a treatment study of patients with PC19S is feasible in general practice. It might also reveal prednisolone and/or vitamin B1, B6 and B12 as an effective and safe therapy to alleviate symptoms of PC19S.

### Electronic supplementary material

Below is the link to the electronic supplementary material.

Supplementary Material 1

## Data Availability

The datasets used and/or analysed during the current trial are available from the corresponding author on reasonable request. All de-identified data can be shared upon reasonable request to the corresponding author as soon as the trial will be completed and the results will have been published. A shortened version of the protocol has already been provided to the public, healthcare professionals and patients via the project homepage www.previtacov.de. Trial results will be published via the trial homepage, too, but first in national and international scientific journals.
